# Psychotropic Drugs Levels in Seminal Fluid: A New Therapeutic Drug Monitoring Analysis?

**DOI:** 10.3389/fendo.2021.620936

**Published:** 2021-03-11

**Authors:** Rossella Mazzilli, Martina Curto, Donatella De Bernardini, Soraya Olana, Matilde Capi, Gerardo Salerno, Fabiola Cipolla, Virginia Zamponi, Daniele Santi, Fernando Mazzilli, Maurizio Simmaco, Luana Lionetto

**Affiliations:** ^1^Andrology Unit, Department of Clinical and Molecular Medicine, Sant’Andrea Hospital, Sapienza University of Rome, Rome, Italy; ^2^International Consortium for Mood Psychotic and Mood Disorders Research, McLean Hospital, Belmont, MA, United States; ^3^Department of Mental Health, ASL Roma 3, Centro di Salute Mentale XI Municipio, Rome, Italy; ^4^Spectrometry-Clinical Biochemistry Laboratory, Sant'Andrea University Hospital, Rome, Italy; ^5^Department of Neurosciences, Mental Health & Sensory Organs (NESMOS), Sapienza University of Rome, Rome, Italy; ^6^Department of Biomedical, Metabolic, and Neural Sciences, University of Modena and Reggio Emilia, Modena, Italy

**Keywords:** psychotropic drugs, liquid-chromatography-mass-spectrometry, LC-MS/MS, metabolomics (OMICS), male infertility, antidepressant, antipsychotics

## Abstract

The aim of this observational study was to develop a new quantitative liquid chromatography-mass spectrometry (LC-MS/MS) method for Therapeutic-Drug-Monitoring (TDM) of psychotropic drugs in seminal fluid to investigate potential gonadotoxic effects in patients with reduced fertility. After the validation of the LC-MS/MS method for psychotropics’ levels determination in seminal fluid, we included 20 male partners of infertile couples with idiopathic and/or unexplained male infertility, treated with psychotropic medications for more than 3 months and 10 untreated fertile controls. General and andrological clinical examination, semen analysis and seminal drugs, and metabolites levels determination were performed for each subject. Of the 20 patients included, 6 were treated with antidepressants; 4 with benzodiazepines and 10 with antipsychotics. Seminal drugs and metabolites levels were detectable in all samples. In particular, alprazolam, olanzapine, and levetiracetam showed seminal and serum similar concentrations, while fluoxetine, quetiapine, and aripiprazole were detectable, but seminal levels were significantly lower than the serum therapeutic range. Sperm progressive motility was significantly reduced in subjects treated with psychotropic drugs compared to the untreated controls (p = 0.03). Sperm concentration and progressive motility were significantly reduced in subjects treated with antipsychotics compared to the untreated controls and to the other classes of psychotropics (p < 0.05). In conclusion, this study reports a validated LC-MS/MS method for the detection of seminal psychotropic levels and preliminary data suggesting a potential correlation of seminal psychotropics with alterations of sperm concentration and motility. Pending larger studies, semen TDM might represent a new pivotal tool in the clinical management of reduced fertility in males treated with psychotropic medications.

## Introduction

Infertility, which is defined as the failure to obtain a clinical pregnancy after at least 12 months of regular unprotected sexual intercourses, can be due to male factors in about 30%–50% of cases, alone or in combination with female factors (1). The effects of many drug classes (i.e., chemotherapeutic agents, antihypertensive drugs, 5α-reductase inhibitors, antibiotics, and psychotropics) on male reproductive system have been evaluated in several studies with inconclusive results (2, 3).

Psychotropic drugs are largely used worldwide and frequently prescribed to patients, representing some of the most used drugs with prescription prevalence in the general population ranging from 6% to 15% (4, 5). Several classes of psychotropics have been associated with reduced male fertility, mainly due to three pathophysiological mechanisms: (a) increased prolactin and gonadotropins levels with testosterone reduction; (b) sexual dysfunction, primarily affecting erection and ejaculation; and (c) semen quality alteration (2, 3, 6, 7). Although the hormonal changes and the sexual dysfunction may affect sperm quality, a direct spermicidal activity and other toxic effects of psychotropics have been reported *in vitro* for several classes, such as antidepressants [selective serotonin inhibitors (SSRIs), tricyclics (TCA), noradrenaline and dopamine reuptake inhibitors (NDRI)] and mood stabilizers (carbamazepine, sodium valproate, lithium) (3, 8–11). Accordingly, further studies in animals and humans treated with psychotropic drugs reported seminal fluid changes, such as reduced sperm quality or increased DNA alterations (12, 13). Hormonal and sexual side effects of psychotropics occur relatively frequently and are directly associated to their mechanism of action, while their potential gonadotoxic effects still remain unclear. Gonadal toxicity might be partially linked to hormonal and sexual changes induced by many psychiatric drugs. However, several *in vitro* and *in vivo* studies suggest a direct spermatozoa-damaging effect related to psychotropics (7–10).

Therapeutic drug monitoring (TDM) represents a metabolomic tool able to assess the actual phenotype of drug metabolites in several biological matrices and clinical settings. In particular, TDM might be applied to drug levels determination in seminal fluid and therefore contribute to the study of the role of psychotropics in male infertility (14). Although several analytical TDM methods have been validated in different biological matrices, such as serum, plasma, urine, cells and tissues, only few drug assays have been developed in seminal fluid (i.e., antiretrovirals, chemotherapics, and antibiotics). In addition, data on seminal drug levels and their relationship with semen features, possibly useful to investigate psychotropics gonadotoxic effects in humans, are not available in literature.

Therefore, the aim of this study was to develop a new analytical method for psychotropic drugs determination in seminal fluid of patients suffering from reduced fertility of unknown cause (i.e., idiopathic male infertility) and to evaluate their relation with semen alterations.

## Materials and Methods

### Patients

This observational, case-control, clinical study included patients referring to the Endocrinology-Andrology Unit of Sant’Andrea Hospital – “Sapienza” University of Rome. Eligible patients were invited to participate in the study and, in case of acceptance, were informed about the purposes of the study and signed a written consent. Inclusion criteria were: (a) male partner of infertile couples as defined by WHO criteria, (b) treated with any psychotropic drugs from at least three months before the enrolment, (c) levels of FSH, LH, E2, testosterone, and prolactin within the reference ranges. Exclusion criteria were: (a) use of any other medications and/or nutraceutics, (b) any known cause of infertility, such as previous cycles of chemo- or radiotherapy, traumas, orchitis, funicular torsions and cryptorchidism, genetic alterations, hyper- or hypogonadotropic hypogonadism, hypo-/hyperthyroidism, hyperprolactinemia, obstructive azoospermia, severe varicocele, and other possible andrological diseases with negative impact on spermatogenesis.

Moreover, 10 healthy age-matched men, who have conceived in the previous 6 months, were included in the Control Group. None of them used psychotropic drugs.

All patients included in the study were evaluated as follows: *(i)* andrological clinical examination: testis (shape, size, and appearance), epididymis, penis, and body hair distribution, possible presence of gynecomastia; *(ii)* recording of demographic characteristics and Body Mass Index (BMI) calculation; *(iii)* recording of use of any kind of medication.

### Semen Analysis

Seminal fluid of each participant was collected by masturbation after sexual abstinence between 2 and 7 days. Semen analysis was carried out according to 2010 WHO guidelines, evaluating spermatozoa morphology, motility, and concentration (1).

The sample container was placed in an incubator (37°C) for 30–60 min. The physical and chemical characteristics of the seminal liquid were then evaluated (appearance, pH, liquefaction, viscosity).

The seminal parameters were then evaluated under the microscope (sperm concentration, percentage of motility, and sperm morphology). In particular, a Superimposed Image Analysis System (SIAS) software (Delta Sistemi, Rome, Italy), a validate method based on superimposed images, was used to assess sperm motility (15, 16). The system can superimpose six sequential frames onto a monitor producing a final image of a complete series of superimposed frames allows the evaluation of the percentage of motile spermatozoa and their kinetic characteristics.

According to WHO (5° percentile), we considered: i) oligozoospermia: total number of spermatozoa below the lower reference limit (15 × 10^6^/ml); ii) asthenozoospermia: percentage of progressively motile spermatozoa below the lower reference limit (32%); iii) teratozoospermia: percentage of morphologically abnormal spermatozoa above the reference limit (96%) (1).

### Liquid Chromatography-Mass Spectrometry (LC-MS/MS) Analysis

#### Chemicals and Reagents

Pure compounds of Fluoxetine, Quetiapine, Olanzapine, Levetiracetam, Aripiprazole and Alprazolam, and internal standard (IS) dansyl-norvaline were purchased from Sigma Aldrich (St Louis, MO). HPLC-grade acetonitrile was purchased from Carlo Erba reagents (Milan, Italy) and formic acid was from Merck (Darmstadt, Germany). Water was deionized and filtered by means of a Milli-Q Plus equipment (Millipore Corporation, Bedford, MA).

#### Stock Solutions and Working Standard

Stock solutions (1 mg/ml) of Fluoxetine, Quetiapine, Olanzapine, Levetiracetam, Aripiprazole, and Alprazolam were prepared by dissolving the pure analytes in the specific solvent depending on their solubility. The working solutions were prepared by diluting stock solutions with deionized water to obtain a final concentration of 250 ng/ml for Fluoxetine, Quetiapine, Olanzapine, Levetiracetam, Aripiprazole, and Alprazolam. All solutions were stored at −20°C until use and then the calibration curves for each analytes were obtained by serial dilution of the highest concentration calibration standard solution, according to the therapeutic serum concentration range of each compound. Calibrator samples and QC samples were treated exactly as patients’ specimens.

#### Sample Preparation

Semen samples were centrifuged at 1,500g for 10 min and an aliquot of 500 µl of seminal plasma were stored at −20°C until processing. Forty µl of QCs, patient samples or calibrators were added to 160 µl of acetonitrile solution for serum deproteinization. The samples were vortex and mixed for 10 s and centrifuged at 14,000 g for 10 min. Forty microliters of clean upper layer were diluted in a vial with 160 µl of 0.1% aqueous formic acid and vortexed for 5 s. Fifteen microliters were injected into the chromatographic system.

#### Chromatographic Conditions

The HPLC analysis was performed using an Agilent Liquid Chromatography System series 1100 (Agilent Technologies, USA) which included a binary pump, an auto-sampler, a solvent degasser, and a column oven. Chromatographic separation was performed using a reversed phase column (Kynetex ^®^ 2.6 µm C18 100 Å pore size, LC Column 50 x 2.00, mm, Phenomenex, CA, USA) equipped with a security guard precolumn (Phenomenex, Torrance, CA, USA) containing the same packing material. The mobile phase consisted of a solution of 0.1% aqueous formic acid (eluent A) and 100% acetonitrile (eluent B); elution was performed at flow rate of 400 μl/min and eluted with a linear gradient from 10% to 90% acetonitrile. The oven temperature was set at 40°C. The injection volume was 20 μl, and the total analysis time was 15 min.

#### Mass Spectrometry Conditions

The mass spectrometry method was performed on a 3200 triple quadrupole system (Applied Biosystems, Foster City, CA, USA) equipped with a Turbo Ion Spray source. The detector was set in the positive ion mode. The ion spray voltage was set at 5,000 V and the source temperature was 300°C. The collision activation dissociation gas was set at medium value and nitrogen was used as collision gas. The Q1 and Q3 quadrupoles were tuned for the unit mass resolution. The transitions of the precursor ions to the product ions were monitored with a dwell time of 100 ms for each analyte. The instrument was set in the multiple reaction monitoring mode. For each analyte, two transitions were selected–the most intense as quantifier and the less intense as qualifier. Mass spectrometer parameters were optimized to maximize sensitivity for all analytes (**Table 1**). Data were acquired and processed with Analyst 1.5.1 software.

**Table 1:** Mass spectrometry parameters.

Analyte	Precursor ion (*m/z*)	Fragment (*m/z*)	DP (V)	EP (V)	CE (V)	CXP (V)
**IS**	351.1	170.0	42.0	7.0	26	2.6
**Fluoxetine**	310.0	259.2 148.0 117.3	40.0 40.0 40.0	9.4 9.4 9.4	21.0 10.0 64.0	3.8 2.3 3.2
**Quetiapine**	383.9	279.0 252.9 220.8	49.5 49.5 49.5	5.1 5.1 5.1	30.1 33.7 48.7	3.7 3.3 2.4
**Olanzapine**	313.2	282.1 255.9	43.8 43.8	8.8 8.8	30.9 27.0	2.5 2.0
**Levetiracetam**	171.2	154.2 126.2	21.1 21.1	2.8 2.8	14.9 18.0	2.1 2.9
**Aripiprazole**	448.1	243.1 217.8	53.0 53.0	9.4 9.4	41.0 31.0	3.2 2.4
**Alprazolam**	309.1	281.1 274.2 205	53.0 53.0 53.0	11.0 11.0 11.0	26 38.8 59.2	7.2 4.9 2.6

IS, internal standard; DP, declustering potential; EP, entrance potential; CE, collision energy; CXP, collision cell exit potential.

#### Method Validation

Processed calibration standards and QC samples were used to develop the calibration curve for the method validation. The validation was conducted considering selectivity, LLOQ, recovery, accuracy and precision, matrix effects, and stability. This method was validated following the European Medicines Agency Guideline on bioanalytical method validation.

#### Specificity, Matrix Effect, and Carry-Over

No interference was observed at the retention times of all the drugs. The blank seminal plasma used for this study was free from drugs at the retention times of the analytes. The matrix effect was calculated using the ratio of the analyte area spiked in the blank seminal plasma after sample treatment to the analyte area in a working standard solution. All samples were confirmed not to show CVs over 15%. Carry-over was assessed considering the peak area of each compound in a black sample analyzed after the injection of 10 μg/ml standard solution. The peak areas were found to be lower than 20% of the peak area of the LLOQ sample.

#### Sensitivity and Linearity

LLOQ were 1.0 ng/ml for Fluoxetine, 2.5 ng/ml for Quetiapine, 5 ng/ml for Olanzapine, 1.5 ng/ml for Levetiracetam, 2.5 ng/ml for Aripiprazole, and 2.0 ng/ml Alprazolam. These values were considered as adequate, since they allowed low concentrations of the compounds to be detected (17). The linear regression of the calibration curves for each analyte showed regression coefficients >0.998 in all cases.

#### Accuracy and Precision

The accuracy results ranged from 90.4 to 104.5% and from 88.6 to 108% for the intraday and interday analysis, respectively. The precision data (%CV) showed that all the concentrations of each QC sample analyzed were better than 10% for all analytes over the respective LLOQs.

#### Recovery and Stability

The analytical method used in this study reported a mean recovery higher than 86.2% for all the compounds. Recovery levels were found to be consistent for each drug over its respective calibration range, which indicated that the extraction efficiency is not influenced by the concentration in the ranges analyzed. Long-term stability of the compounds, after 60 days at −80°C, as well as after three freeze (−20°C)/thaw (24°C) cycles, was also confirmed.

### Statistical Analysis

Continuous variables were reported as mean ± standard deviation. Rates or categorical parameters were reported as numbers and percentages. Between-groups differences were assessed using the paired t-test for continuous data and correlation with Spearman’s rho. P < 0.05 was considered statistically significant.

Statistical analysis was carried out with GraphPadInStat software (Version 3.06 for Windows, San Diego, CA, USA).

Based on previous observations (1, 4, 5), we assumed a male factor in 40% of the infertile couples and a prevalence of 10% in the general population using psychotropic drugs. Therefore, the recruitment of 16 participants would be required to achieve a power of 80%, with an estimated α error of 0.05 and β error of 0.2. The sample size was calculated with clincalc.com.

## Results

A total of 41 infertile patients treated with psychotropic medication were evaluated. Of them, 21 patients were excluded, 10 were taking nutraceutics or also non-psychotropic medications, 2 presented hypogonadism, 2 were affected by hyperprolactinemia, and 1 by hypothyroidism, 3 had severe varicocele, 1 had a previous funicular torsion, 1 had a previous testicular trauma, and 1 had obstructive azoospermia. Thus, the final sample size included 20 patients. None of the subjects suffered from sexual dysfunction or ejaculatory dysfunction.

The mean age of patients enrolled was 34.8 ± 3.7 years in the case group and 34.1 ± 3.8 in the control group (p = 0.64). All of them were Caucasian.

The mean BMI of the case and of the control groups were 22.5 ± 1.1 kg/m^2^, ranging between 20.8 and 24.1 kg/m^2^, and 22.2 ± 1.5 kg/m^2^, ranging between 19.9 and 23.9 kg/m^2^, respectively (p = 0.51). No differences were detected between cases and controls considering objective anomalies of testis, epididymis, penis, and body hair distribution or gynecomastia.

### Medications

According to the class of psychotropics taken, the subjects enrolled in the case group were divided in three study groups: Group A: n. 6/20 (30.0%) subjects treated with SSRI antidepressants (Fluoxetine); Group B: n. 4/20 (20.0%) subjects treated with benzodiazepines (Alprazolam); Group C: n. 10/20 (50.0%) subjects treated with antipsychotics/antiepileptics (Quetiapine, Olanzapine, Levetiracetam, Aripiprazole). The treatment duration ranged between 10 and 24 months.

**Figure 1:**
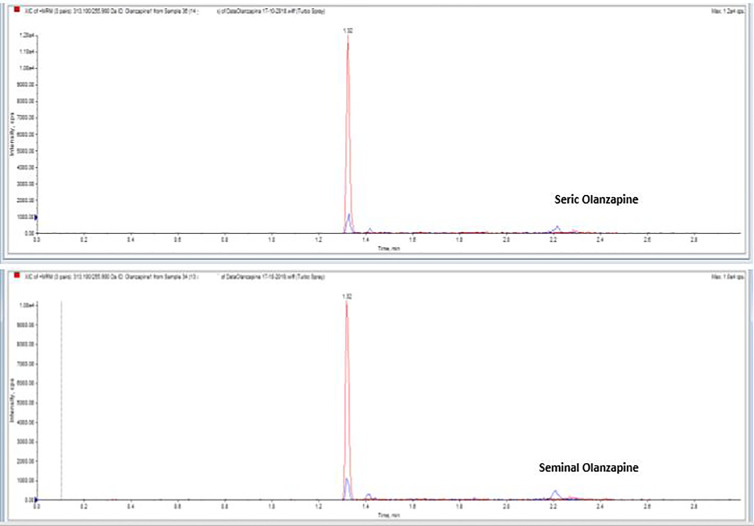
Serum and seminal plasma concentration of Olanzapine with liquid chromatography tandem mass spectrometry.

Drugs and their metabolites levels were detected in serum as well as in seminal fluid of the case group patients (**Table 2**). In particular, Alprazolam, Olanzapine (**Figure 1**), and Levetiracetam were within the normal range in serum and showed similar concentrations in semen, while Fluoxetine, Quetiapine, and Aripiprazole were detectable in semen, but their concentrations were lower than the serum ones. A Pearson correlation test highlighted a correlation between Fluoxetine concentration and sperm number reduction, both concentration/ml and total sperm number (p < 0.5; r = 0.8).

**Table 2:** Seminal and serum concentrations of psychotropic drugs.

Psychotropic		Cases (n.)	Serum normal range	Serum concentration	Seminal concentration
**SSRI**	**Fluoxetine**	6	120–500 ng/ml	266.2 ± 59.8 ng/ml	27.5 ± 16.3 ng/ml
**Benzodiazepines**	**Alprazolam**	4	5–50 ng/ml	17.8 ± 10.8 ng/ml	9.1 ± 8.1 ng/ml
**Antipsychotics/Antiepileptics**	**Quetiapine**	3	100–500 ng/ml	318.0 ± 76.7 ng/ml	34.3 ± 14.9 ng/ml
	**Olanzapine**	2	20–80 ng/ml	42.0 ± 19.8 ng/ml	37.4 ± 21.3 ng/ml
	**Levetiracetam**	2	10–37 µg/ml	20.5 ± 2.1 µg/ml	10.8 ± 0.4 µg/ml
	**Aripiprazole**	3	150–500 ng/ml	240.3 ± 62.3 ng/ml	39.1 ± 17.2 ng/ml

Data are presented as mean +/−SD.

In the control group, none of the drugs and their metabolites were detected in serum and in seminal fluid.

### Semen Parameters

Semen parameters are described in **Table 3**. Overall, sperm progressive motility was significantly reduced in patients treated with psychotropic drugs (Total Group) compared to the Control Group (36.8 ± 11.6% vs. 46.3 ± 10.0%, p = 0.03). No significant differences between Total Group and Control Group were observed in the other parameters (volume 3.2 ± 1.1 ml vs. 3.2 ± 1.3 ml, p = 0.91; concentration: 70.8 ± 30.0 × 10^6^/ml vs. 82.1 ± 17.6 × 10^6^/ml, p = 0.21; total sperm number: 228.4 ± 112.3 × 10^6^ vs. 247.7 ± 99.5 × 10^6^, p = 0.50; abnormal morphology: 73.7 ± 6.9% vs. 75.1 ± 7.7%, p = 0.60).

**Table 3:** Age and seminal profile in subjects treated with psychotropic drugs and in Control Group.

	Group A (n.6)	Group B (n.4)	Group C (n.10)	Total Group (n. 20)	Control Group (n.10)	P value
**Age** ***(years)***	36.3 ± 2.3 (33–39)	33.0 ± 3.7 (29–38)	34.6 ± 4.2 (26–39)	34.8 ± 3.7 (26–39)	34.1 ± 3.8 (27–39)	0.64
**Volume** ***(ml)***	3.0 ± 1.4 (1.5–5.6)	3.9 ± 1.0 (3.1–5.1)	3.1 ± 0.9 (1.4–4.6)	3.2 ± 1.1 (1.4–5.6)	3.2 ± 1.3 (1.6–5.1)	0.91
**Sperm concentration** ***(n*** *×* ***10^6^/ml)***	92.2 ± 23.9 (51–118)	84.3 ± 15.3 (63–98)	52.6 ± 27.4^a,b,c^ (15–98)	70.8 ± 30.0 (15–118)	82.1 ± 17.6 (55–104)	0.21
**Tot. sperm number** ***(n*** *×* ***10^6^/ejac.)***	260.6 ± 72.9 (121.5–330)	406.7 ± 169.0 (195.3–607.6)	167.1 ± 101.5^a,b,c^ (43.4–334-4)	228.4 ± 112.3 (43.4–428.4)	247.7 ± 99.5 (97.6–387.6)	0.50
**Progr. motility** ***(%)***	42.8 ± 11.8 (28–57)	41.3 ± 11.1 (28–55)	31.3 ± 10.0 ^c^ (18–45)	36.8 ± 11.6 (18–57)	46.3 ± 10.0 (18–45)	0.03
**Abnormal morphology *(%)***	74.3 ± 5.9 (66–84)	69.0 ± 5.5 (62–75)	75.1 ± 7.7 (63–88)	73.7 ± 6.9 (62–88)	75.1 ± 7.7 (63–88)	0.60

Data are presented as mean +/−SD (range).

p < 0.05 ^a^ vs. Group A; ^b^ vs. Group B; ^c^ vs. Control Group.

Among the subgroups, sperm concentration and total sperm number were significantly reduced in subjects treated with antipsychotics/antiepileptics (Group C) compared to Group A, Group B, and Control Group (concentration: 52.6 ± 27.4 × 10^6^/ml vs. 92.2 ± 23.9 × 10^6^/ml, vs. 84.3 ± 15.3 × 10^6^/ml and vs. 82.1 ± 16.4 × 10^6^/ml, p = 0.01; total sperm number 167.1 ± 101.5 × 10^6^ vs. 260.6 ± 72.9 × 10^6^, vs. 406.7 ± 169.0 × 10^6^ and vs. 247.7 ± 99.5 × 10^6^; p = 0.01, p = 0.05, and p = 0.05, respectively). Progressive motility was significantly reduced in Group C compared to Control Group (31.3 ± 10.0% vs. 46.3 ± 10.1%, p = 0.003). No differences were observed in volume and abnormal morphology percentage among the three study Groups and the Control Group, as well as for age (**Table 3**).

## Discussion

Side effects of several classes of psychotropic drugs include impaired sexual function and fertility. Many psychotropics hormonal and sexual side effects are directly related to drugs mechanism of action and have been largely studied, mainly due to reduced testosterone and increased prolactin and gonadotropins levels, as well as to the presence of sexual dysfunction, concerning primarily the erection and ejaculation mechanisms. In particular, a direct gonadotoxic effect of some classes of psychotropics has been reported in *in vitro* and *in vivo* studies, both in animals and humans’ models (7), but their action on spermatogenesis and their potential seminal toxicity remains unclear. In fact, only few studies on male infertility have been conducted in patients treated with psychotropic drugs, reporting seminal alterations associated with specific classes of antidepressants, benzodiazepines, and antipsychotics.

In this study, we observed a significant reduction of sperm motility in the Total Group of patients treated with psychotropic drugs compared to the Control Group, and a significantly lower sperm concentration in patients treated with antipsychotics compared to the Control Group and to patients treated with antidepressants or benzodiazepines. No significant differences were observed in volume and sperm morphology. Our results are partially in line with those of Asadi-Pooya and Colleagues (2015), who investigated the effects of carbamazepine on semen parameters and observed impaired sperm concentration, progressive motility, and morphology (18). Similarly, Ocek and Colleagues (2018), reported that semen volume and number of typical forms were significantly lower in patients treated with antiepileptics (carbamazepine or sodium valproate) compared to the controls (19). Regarding antidepressants, Tanrikut and Colleagues (2010) reported that paroxetine induced abnormal sperm DNA fragmentation in men with normal semen parameters, without other seminal effects (12). Similarly, Safarinejad and Colleagues (2008) observed an impairment on semen quality and a damage on sperm DNA in patients receiving SSRI (10).

However, a direct toxic activity on spermatozoa might be only hypothesized. Therefore, in our study, a new TDM analytical method for the determination of psychotropics in seminal fluid has been developed, in order to demonstrate the association between drugs presence and spermatozoa impaired features. TDM consist in measuring specific drugs at designated intervals to maintain a constant concentration to optimize individual dosage regimens. It is mainly used for monitoring drugs with narrow therapeutic ranges, marked pharmacokinetic variability and adverse effects (20).

This method is also used for oncological therapy (21), anti-infective (22), and psychotropics treatment (23).

To our knowledge, no other studies are available which are detecting both psychotropic drugs and their metabolites in seminal fluid of infertile men. In fact, although several analytical TDM methods have been validated in different biological matrices (i.e., serum, plasma, urine, cells, and tissues), few drug assays have been developed for seminal fluid. Antiretrovirals, chemotherapics, and antibiotics are the most studied drugs in seminal fluid with gonadotoxic effects. For example, Lowe and Colleagues (2007) observed that both didanosine and tenofovir penetrated into seminal plasma and a reduction of progressively motile sperm in patients treated with these antiretrovirals (24).

Based on our preliminary results in a restricted group of treated patients, a minority of the studied medications seems to be transported in the seminal fluid in low concentrations and might be related to reduced fertility in male partners of infertile couples. Our study showed a reliable method able to detect and measure the psychotropic drugs in seminal plasma, suggesting their potential impairing effect for semen quality. In particular, it would be important to identify the cut off between “toxic” and “non-toxic” concentration values, as is the case for serum assays (20).

Studies on gonadotoxic effects of psychotropics might potentially affect the indications of psychiatric treatments in adult males looking for a conception by the identification of gonadotoxic severity of the different molecules. Thus, the future evaluation and grading of psychotropic drugs might represent a valid instrument for the clinical assessment and management of infertile couples. Moreover, a lower impact of psychotropic drugs on male fertility might improve patients’ compliance to treatment in a population with a poor adherence high rate.

The major limitations of the study are represented by the restricted sample size and the limited number of psychotropic drugs included. In fact, only few drugs used in clinical practice have been evaluated with the TDM method. Further larger studies should be conducted to clarify the gonadotoxic effects of psychotropics and in particular, of antipsychotics and antiepileptics, as well as the relationship between toxic dose of a certain drug/metabolite to the sperm, the dwell time of drug and sperm which may lead to damage, the resultant sperm damage and its implication on reproductive parameters.

## Conclusions

In conclusion, this study reports a validated LC-MS/MS method for the detection of psychotropic levels in seminal fluid. This new approach shed some light and encourage careful considerations upon a still unclear issue on the management of reduced fertility in males treated with psychotropic medications.

Of course, further larger studies, including additional molecules belonging to this class, are needed, in order to clarify any correlation between seminal psychotropics and sperm alterations, as well as the mechanisms of the gonadotoxic effect.

## Data Availability Statement

The raw data supporting the conclusions of this article will be made available by the authors, without undue reservation.

## Ethics Statement

The studies involving human participants were reviewed and approved by Sapienza University of Rome. Study protocol: RM11916B8900AF26. The patients/participants provided their written informed consent to participate in this study.

## Author Contributions

RM conceived the study, interpreted and analyzed the data, and drafted the manuscript. MCu provided a critical revision of the manuscript. DB, SO, MCa, GS, FC, and VZ acquired and analyzed the data. DS provided a critical revision of the manuscript. FM and MS conceived the study, interpreted and analyzed the data, and provided a critical revision of the manuscript. LL analyzed the data, drafted the manuscript, and provided a critical revision of the manuscript. All authors contributed to the article and approved the submitted version.

## Funding

Sapienza University of Rome. Study protocol: RM11916B8900AF26.

## Conflict of Interest

The authors declare that the research was conducted in the absence of any commercial or financial relationships that could be construed as a potential conflict of interest.
